# Preconception Counseling and Care in the Setting of HIV: Clinical Characteristics and Comorbidities

**DOI:** 10.1155/2015/240613

**Published:** 2015-03-08

**Authors:** Rupsa C. Boelig, Jenell S. Coleman, Jean Keller, Catherine Sewell, Jean Anderson

**Affiliations:** ^1^Department of Obstetrics & Gynecology, Thomas Jefferson University Hospital, Philadelphia, PA 19107, USA; ^2^Division of Gynecologic Specialties, Department of Obstetrics & Gynecology, Johns Hopkins University School of Medicine, Baltimore, MD 21287, USA; ^3^Food and Drug Administration, U.S. Department of Health and Human Services, Silver Spring, MD 20993, USA

## Abstract

*Objective*. To describe the demographic and clinical characteristics of HIV-infected individuals and HIV-affected couples who were referred for preconception counseling (PCC) at a large urban US-based HIV clinic. *Methods*. Electronic medical records were reviewed for HIV-infected individuals and HIV-affected couples. Medical, reproductive, surgical, psychosocial, and family history data were abstracted. Univariate analyses were done. *Results*. There were 8 single HIV-infected women and 100 HIV-affected couples who underwent PCC. HIV-infected women were older (mean age 35 years versus 32 years, *P* = 0.06), were more likely to smoke (23% versus 0%, *P* < 0.01), and had more medical comorbidities (57% versus 33%, *P* = 0.04) than HIV-uninfected women. The majority of couples were serodiscordant (77%), and of these couples, 32% had a detectable plasma viral load and 33% report inconsistent condom use. *Conclusions*. HIV-infected women have a number of medical and psychosocial issues, including those related to HIV that may increase the risk of adverse pregnancy outcomes and HIV perinatal and sexual transmission. PCC is an important intervention to optimize maternal management to improve perinatal outcomes and minimize transmission risks.

## 1. Introduction

Advances in HIV treatment and reductions in perinatal HIV transmission have led to improved quality of life and increased pregnancies among women with HIV [1]. Studies conducted in the era of effective antiretroviral treatment (ART) suggest that HIV-infected women have similar reproductive desires and intentions as HIV-uninfected women [2–4]. Nevertheless, many pregnancies that occur in the setting of HIV are unintended and the most effective forms of reversible contraception are underused [5–8].

Preconception counseling and care (PCC) are associated with improved maternal and fetal outcomes by identifying and modifying risks prior to pregnancy and in preventing unwanted pregnancies [9, 10]. The American College of Obstetricians and Gynecologists and the United States (U.S.) Public Health Services Panel on Treatment of HIV-Infected Pregnant Women and Prevention of Perinatal Transmission recommend offering all HIV-infected women of childbearing age comprehensive PCC as a component of routine primary medical care [11, 12]. The goal of PCC in the setting of HIV is to help HIV-infected individuals and HIV-affected couples make informed reproductive decisions, optimize maternal and paternal health prior to pregnancy, improve pregnancy outcomes, and minimize the risk of vertical and/or horizontal transmission.

Given the lack of data in the literature regarding the clinical characteristics of individuals presenting for PCC in the setting of HIV, we sought to describe the demographic and clinical characteristics of HIV-infected individuals and HIV-affected couples who were referred for PCC at a large urban US-based HIV clinic.

## 2. Materials and Methods

We conducted a retrospective review of electronic medical records of HIV-infected individuals and HIV-affected couples, where at least one of the partners was HIV infected. A convenience sample of patients referred for PCC at the HIV Women's Health Program, which is a comprehensive obstetrics and gynecology (OBGYN) clinical program located within the Johns Hopkins Hospital in Baltimore, Maryland, from January 2002 to January 2013, were included. Patient visits were identified by visits coded for PCC that were entered into an administrative database. For patients who came in for multiple preconception counseling visits, only the first visit was included. The most recently available CD4 cell count and HIV-RNA level at the time of the visit were used. Undetectable plasma viral load (PVL) was defined as <75 copies/mL. Illicit drug use was defined as self-reported use of illegal drugs within the last six months. The Johns Hopkins University Institutional Review Board approved the study.

As part of PCC, a provider with expertise in obstetrics/gynecology and HIV medicine obtained medical, reproductive, surgical, psychosocial, and family history and counseled regarding (1) safer conception, (2) perinatal transmission prevention, (3) use of ART in pregnancy as treatment and/or prophylaxis, and (4) effect of other medical illnesses and medications on pregnancy. If couples wished to defer pregnancy, then highly effective contraceptive options were discussed.

Characteristics of the study population were summarized using mean ± standard deviation (SD) or median and interquartile range (IQR) for continuous variables. Chi-square analysis or Fisher's exact test, as appropriate, was used to compare categorical variables and Wilcoxon rank sum test was used to compare continuous variables. A two-tailed *P* value of <0.05 was considered to be significant. Missing data were excluded from analyses. Stata version 12.0 (StataCorp, Inc., College Station, TX) was used.

## 3. Results

A total of 108 women were seen for PCC during the study period. Of these, 21.3% (23) were in HIV-seroconcordant relationships with HIV-infected men; 51.9% (56) were in HIV serodiscordant relationships with HIV-uninfected men, 19.4% (21) were HIV uninfected in serodiscordant relationships with HIV-infected men, and 7.4% (8) were HIV infected and not currently in a relationship but were interested in pregnancy options. There was a 76% increase in visits for PCC in the last three years reviewed compared to the first three years (data not shown).

### 3.1. Demographics

Mean age was 34.6 years (SD 6.2) and over half of women were 35 years or older (IQR 31–39) (Table 1); there was a trend towards HIV-infected women being older as compared to HIV-uninfected women (35.2 years versus 32.3 years, *P* = 0.06). Over 66% of women were black and, of these, 26% were African. Six percent (6) of women reported active illicit drug use that included marijuana, cocaine, or heroin among HIV-infected women and marijuana alone among HIV-uninfected women. There were significantly more cigarette smokers among HIV-infected women than HIV-uninfected women (23% versus 0%, *P* < 0.01).

### 3.2. Reproductive and Medical History

There were 44% (48) of women who reported no living children and 19% (21) women who reported a history of infertility; however, there were no significant differences between HIV-infected and HIV-uninfected women (*P* = 0.43 and *P* = 0.98, resp.). Exclusive of those who reported infertility, 7% (6) HIV-infected women and 5% (1) HIV-uninfected women reported a prior tubal ligation. More HIV-infected women reported abnormal menstrual cycles compared to HIV-uninfected women (26% versus 14%, *P* = 0.25). In those women who reported previous pregnancies, at least 50% in each group had pregnancy complications (56% HIV-infected versus 50% HIV-uninfected, *P* = 0.74), and among these, most women reported preterm delivery (24% HIV infected versus 20% HIV uninfected, *P* = 0.83).

HIV-infected women were significantly more likely to have at least one additional medical comorbidity, other than HIV, compared to HIV-uninfected women (57% versus 33%, *P* = 0.04). Additionally, though not being statistically significant, there was a greater proportion of HIV-infected women who reported two or more comorbid conditions compared to HIV-uninfected women (29% versus 10%, *P* = 0.07). There was a statistically nonsignificant trend for certain comorbidities to be more common among HIV-infected women compared to HIV-uninfected women including psychiatric illness (20% versus 5%, *P* = 0.10), hepatitis C (10% versus 0%, *P* = 0.13), diabetes (3.5% versus 0%, *P* = 0.4), and hepatitis B (1.2% versus 0%, *P* = 0.6). Hypertension was common (11% versus 14%, *P* = 0.70) among both groups of women.

### 3.3. HIV Clinical Characteristics of Infected Women, Men, and Couples

Although 49% of HIV-infected women had a CD4 count nadir <200 cells/mm^3^, only 5% had a CD4 count <200 cells/mm^3^ at the time of their visit (Table 2). An undetectable PVL was recorded in 70% of women. Combination ART was reported in 80% of women, and among these women, 16% had a detectable PVL and 23% were receiving regimens that contained efavirenz. There was less information available on the HIV-infected male partners. The majority of men (84%) had an undetectable PVL. While 89% of men were receiving combination ART, 13% of these men had a detectable PVL.

Over 75% of the couples were serodiscordant (Table 2). There was a trend towards more HIV-infected women with a detectable PVL among serodiscordant couples compared to seroconcordant couples (35% versus 14%, *P* = 0.09). The proportion of HIV-infected partners on ART was slightly lower among serodiscordant couples compared to seroconcordant couples (80% versus 86%, *P* = 0.61). Moreover, HIV serodiscordant couples were significantly more likely to “always use” condoms (45 (67%) serodiscordant couples versus 5 (23%) seroconcordant couples), while HIV seroconcordant couples were more likely to “never or inconsistently use” condoms (22 (33%) serodiscordant couples versus 17 (77%) seroconcordant couples, *P* < 0.001). Twenty-four percent of HIV serodiscordant couples who reported “never or inconsistently use” condoms had a detectable PVL.

## 4. Discussion

To our knowledge, this is the first report detailing the clinical characteristics of HIV-infected individuals and HIV-affected couples who sought PCC in a high resource setting. Women were predominantly black, reflecting national and local epidemiology of HIV in women [13]. Our findings highlight several important considerations in PCC of HIV-infected and HIV–affected couples.

First, a primary goal of PCC in the setting of HIV is to achieve maximal viral suppression before conception. Detectable HIV PVL and lack of effective ART have been directly associated with perinatal and sexual transmission [12]. Thus, adherence to ART should be optimized. Although a majority of HIV-infected women achieved maximal viral suppression at the time of their PCC visit in our study, improvements can be made. Among serodiscordant couples, over a third had detectable viremia, and of these viremic couples, almost 25% reported inconsistent condom use.

Second, there are a number of options for safer conception. Serodiscordant couples with an HIV-infected female partner may consider home insemination during the most fertile period of the menstrual cycle using a needleless syringe, while continuing consistent condom use. Serodiscordant couples with an HIV-infected male partner may consider assisted reproductive technology using sperm washing with intrauterine insemination or in vitro fertilization, although these methods are often limited by access and cost. In addition to ART for treatment of the HIV-infected partner, preexposure prophylaxis (PrEP) for the uninfected partner should be discussed [14], and timed condomless sex can be considered. However, condomless sex in the setting of nonmonogamy might also increase the risk of acquisition of other sexually transmitted infections (STI). Therefore, it is critical to discuss sexual partnerships and screen partners for STIs.

Third, one of the preferred ART regimens for antiretroviral-naïve adults is efavirenz/tenofovir/emtricitabine in a coformulation given as one pill once-daily, based on its virologic efficacy, safety, tolerability, and convenience. However, efavirenz is the only antiretroviral agent with documented teratogenic potential, although that risk may be lower than that previously anticipated [15]. Current US guidelines, however, still recommend an alternative regimen in women planning to conceive and avoidance of efavirenz-containing regimens in the first 8 weeks of pregnancy [12].

Fourth, there was a high frequency of additional comorbidities, many of which may have independent adverse effects on pregnancy outcomes. Depression and other psychiatric illnesses have been associated with lower rates of ART adherence and optimal viral suppression [16]. Hypertension is associated with intrauterine growth restriction and preeclampsia [17]. In addition, providers should be aware of medications used to treat medical illnesses that may be teratogenic and require additional counseling and/or a change to a safer alternative prior to conception.

Fifth, approximately 20% of women reported tobacco use, which has been associated with up to twofold increased risk of low birth weight, preterm birth, and infants for gestational age among HIV-infected mothers [18]. Use of alcohol and illicit drugs, though being reported infrequently by women in this study, is associated with adverse pregnancy outcomes including congenital anomalies, preterm delivery, abruption, and growth restriction [19]. Thus, it is important to assess and encourage cessation of tobacco and illegal drug use prior to conception.

Our study has limitations. First, it is a small convenience sample of patients engaged in care at a single HIV program at a US institution, which may limit the generalizability. Second, our sample included couples that were referred by their HIV providers when a desire for childbearing was expressed or elicited or prompted by concerns regarding ART safety in pregnancy. It is possible that some HIV providers provided PCC themselves and did not refer to our program. However, a prior study in this same hospital found that personalized discussions about pregnancy between the woman and her HIV provider occurred in only one-third of women, two-thirds of which were initiated by the woman herself [20]. In fact, the number of patients referred for PCC in our clinic steadily increased over time, likely reflecting greater awareness of the effectiveness of ART for maternal health and longevity and prevention of sexual and perinatal transmission. Additionally, it is possible that some study subjects were not included due to miscoding of the visit diagnosis. Last, there were missing data, which may have limited our ability to discern significant differences.

## 5. Conclusion

Overall, our study demonstrates that HIV-infected individuals and HIV-affected couples have a number of medical and psychosocial issues, including those related to HIV, that may increase the risk of adverse pregnancy outcomes and HIV perinatal and sexual transmission. Studies show that HIV-infected women often report a desire for future childbearing, but discussions with providers often do not take place and, when they do, are more likely to be patient-initiated and may involve judgmental provider attitudes [21–23]. PCC is an important intervention to counsel and optimize HIV and other medical conditions to minimize sexual risks and improve perinatal outcomes.

## Figures and Tables

**Table 1:** Demographics of women by HIV status.

	HIV infected (*n* = 87)	HIV uninfected (*n* = 21)	Total (*n* = 108)
Race			
African-American	55% (48)	29% (6)	50% (54)
African	20% (17)	9.5% (2)	17% (19)
Caucasian	17% (15)	38% (8)	21% (23)
Hispanic	3.5% (3)	9.5% (2)	5% (5)
Asian	3.5% (3)	0% (0)	3% (3)
Not reported	1% (1)	14% (3)	4% (4)
Age			
Mean (SD)	35.2 (6.3)	32.3 (5.2)	34.6 (6.2)
Median (IQR)	35 (31–40)	34 (29–36)	35 (31–39)
35 years or older	55% (48)	38% (8)	52% (56)
Substance use^*^			
Illicit drug use	5% (4)	10% (2)	6% (6)
Current tobacco use	23% (20)	0% (0)	19% (20)
Heavy alcohol use (>7 drinks/week)	2% (2)	0% (0)	2% (2)
HIV transmission risk			
Heterosexual sex	71% (62)	—	—
Injection drug use	11.5% (10)	—	—
Heterosexual sex or injection drug use	2% (2)	—	—
Blood transfusion	3.5% (3)	—	—
Unknown	11% (10)	—	—
Male partner HIV status			
HIV infected	26.4% (23)	100% (21)	—
HIV uninfected/unknown	64.4% (56)	—	—
No partner	9.2% (8)	—	—

Data are % *n* otherwise stated.

^*^Complete data available for 104 participants.

—: not applicable.

**Table 2:** Clinical characteristics of HIV-infected women, HIV-infected men, and HIV-affected couples.

	HIV infected women (*n* = 87)	HIV infected men (*n* = 44)	HIV serodiscordant couples (*n* = 77)			HIV seroconcordant couples (*n* = 23)		P^**♮**^
	F	M	F	M	Either	F	M	
Years living with HIV								
*n*	*87 *	*28 *	*55 *	*20 *	*75 *	*21 *	*8 *	.51
Mean (SD)	7 (5.7)	6.1 (6.1)	7 (5.8)	4.9 (4.4)	—	8 (6.4)	9 (87.7)	
CD4 count, cells/mm^3^								
*n*	*83 *	*28 *	*56 *	*18 *	*74 *	*21 *	*10 *	.18
<200	5 (4)	11 (3)	7 (4)	5 (1)	7 (5)	0 (0)	20 (2)	
200–499	46 (38)	64 (18)	39 (22)	67 (12)	46 (34)	60 (12)	60 (6)	
≥500	49 (41)	25 (7)	54 (30)	28 (5)	47 (35)	40 (8)	20 (2)	
CD4 nadir, cells/mm^3^								
*n*	*76 *	*18 *	*50 *	*16 *	*66 *	*21 *	*2 *	.06
<200	49 (37)	56 (10)	42 (21)	50 (8)	44 (29)	67 (14)	100 (2)	
≥200	51 (39)	44 (8)	58 (29)	50 (8)	56 (37)	33 (7)	0 (0)	
Plasma viral load, copies/mL								
*n*	*81 *	*31 *	*54 *	*19 *	*73 *	*21 *	*12 *	.07
<75	70 (57)	84 (26)	65 (35)	79 (15)	68 (50)	86 (18)	92 (11)	
75–999	9 (7)	6.5 (2)	13 (7)	5 (1)	11 (8)	0 (0)	8 (1)	
1,000–9,999	9 (7)	3 (1)	13 (7)	5 (1)	11 (8)	0 (0)	0 (0)	
≥10,000	12 (10)	6.5 (2)	9 (5)	11 (2)	10 (7)	14 (3)	0 (0)	
Undetectable plasma viral load (<75 copies/mL)								
*n*	*81 *	*31 *	*54 *	*19 *	*73 *	*21 *	*12 *	.09
Yes	70 (57)	84 (26)	65 (35)	79 (15)	68 (50)	86 (18)	92 (11)	
No	30 (24)	16 (5)	35 (19)	21 (4)	32 (23)	14 (3)	8 (1)	
Antiretroviral therapy								
*n*	*87 *	*38 *	*56 *	*20 *	*76 *	*23 *	*18 *	.76
Currently on ART	80.5 (70)	89 (34)	79 (43)	90 (18)	80 (61)	84 (19)	89 (16)	
Efavirenz regimen^#^	23 (16)	—	21 (9)	—	—	26 (5)	—	—

Data in table are % (*n*) unless otherwise stated.

Denominator for each variable (*n*) is listed due to missing data.

SD: standard deviation; ART: antiretroviral therapy; —: n/a; F: female patient; M: male patient.

^*♮*^Wilcoxon rank sum test was used for comparison of means, and Fisher's exact or Pearson chi square was used for comparisons of categorical variables among women in serodiscordant versus seroconcordant relationships.

^#^Efavirenz regimen percentage is limited to women who are taking ART.
